# Inactivation of Ricin Toxin by Nanosecond Pulsed Electric Fields Including Evidences from Cell and Animal Toxicity

**DOI:** 10.1038/srep18781

**Published:** 2016-01-05

**Authors:** Kai Wei, Wei Li, Shan Gao, Bin Ji, Yating Zang, Bo Su, Kaile Wang, Maosheng Yao, Jue Zhang, Jinglin Wang

**Affiliations:** 1 State Key Joint Laboratory of Environmental Simulation and Pollution Control, College of Environmental Sciences and Engineering, Peking University, Beijing 100871, China; 2 Academy for Advanced Interdisciplinary Studies, Peking University, Beijing 100871, China; 3 Institute of Microbiology and Epidemiology, Academy of Military Medical Sciences, Beijing 100071, China; 4 College of Engineering, Peking University, Beijing 100871, China

## Abstract

Ricin is one of the most toxic and easily produced plant protein toxin extracted from the castor oil plant, and it has been classified as a chemical warfare agent. Here, nanosecond pulsed electric fields (nsPEFs) at 30 kV/cm (pulse durations: 10 ns, 100 ns, and 300 ns) were applied to inactivating ricin up to 4.2 μg/mL. To investigate the efficacy, cells and mice were tested against the ricin treated by the nsPEFs via direct intraperitoneal injection and inhalation exposure. Results showed that nsPEFs treatments can effectively reduce the toxicity of the ricin. Without the nsPEFs treatment, 100% of mice were killed upon the 4 μg ricin injection on the first day, however 40% of the mice survived the ricin treated by the nsPEFs. Compared to injection, inhalation exposure even with higher ricin dose required longer time to observe mice fatality. Pathological observations revealed damages to heart, lung, kidney, and stomach after the ricin exposure, more pronounced for lung and kidney including severe bleeding. Sodium dodecyl sulfate polyacrylamide gel electrophoresis(SDS-PAGE) and circular dichroism (CD) analyses revealed that although the primary structure of ricin was not altered, its secondary structures (beta-sheet and beta-turn) underwent transition upon the nsPEFs treatment.

Ricin toxin (RT) is one of the most toxic and easily produced plant protein toxins extracted from the castor oil plant-*Ricinis communis*^1^,^2^. It is estimated that 50,000 tons of RT are produced annually as a by-product of castor oil^3^. The RT has a heterodimeric structure consisting an A-chain (approximately 30 kDa, the toxic component) and B-chain (approximately 32 kDa) linked together by a disulfide bond^4^. In general, the B-chain facilitates internalization of the A-chain, subsequently the A-chain exerts its toxic effects by inhibiting protein synthesis leading to cell death^4^. The degree of health damage by the ricin is dependent on both the amount of toxin internalized and also the route of exposure^4^,^5^. After inhaling the ricin aerosol particles, respiratory failure and allergic response can be triggered for human and mice^6^,^7^. The median lethal dose (LD_50_) of inhaling exposure for mouse was described to be approximately 3–5 μg/kg of ricin particles of less than 5 μm^7^. In contrast, uptake of food and water contaminated with ricin by humans and animals can lead to the circulatory collapse and subsequently their death^8^. The LD_50_ of ingestion ricin is 30 mg/kg for mouse according to experiments and 1–20 mg/kg for human beings by estimation^7^. Owing to its ease of production and high toxicity, the ricin has been, as early as 1918, listed as one of chemical warfare agents by US Department of Defense^7^, and the ricin toxin was used to develop biological weapon during World War I and World War II^9^.

Now, the ricin toxin is increasingly being considered by terrorist groups to carry out bio-chemical attacks on high profile individuals and buildings. For example, in 2003, a terrorist threatened to contaminate the water supply using ricin if his demands failed to be satisfied in South Carolina, US^10^. One year after, a letter with ricin powder addressed to the White House was discovered in the mailroom serving US Senator office in South Carolina^10^. On April 16^th^ and 17^th^, 2013, two letters were mailed out from Memphis, Tennessee, US. The first one was addressed to the office of Mississippi Republican Senator, and the second one was mailed to the President of the United States^11^. Both of the mails contained white powder which was later tested positive for ricin toxin, provoking a national security alert. Most recently, the terrorist groups also threatened to carry out terrorist attacks using ricin in the United Sates^12^. A ricin toxin attack by terrorist groups could cause not only human death but also significant public fear. As a consequence, practical solutions to combat the ricin threat are urgently needed for the sake of national security and the public welfare.

Over the years, a range of methods have been attempted, including heating, chemicals^13^, and vaccination^3^,^10^. However, these methods fall short of providing a satisfactory solution toward the ricin threat or not validated against humans. In recent decades, nanosecond pulsed electric fields, known as nsPEFs, is attracting wide attention in practical applications of medicine and biology, including calcium fluctuation^14^,^15^, changes in membrane permeability^16^,^17^,^18^,^19^ and voltage change^20^,and even solid tumor treatment^21^. The nsPEFs approach delivers electric pulses with low, non-thermal energy, but instantaneous high power for ultra-short durations (nanoseconds) and high electric fields (tens kV/cm). Due to the ultra-short duration of pulses, nsPEFs can also induce a series of unique biological effects such as apoptosis^22^,^23^,^24^,^25^, and phosphatidylserine translocation^26^,^27^,^28^ but not through the hyperthermal effects. Cell apoptosis induced by the nsPEFs has been observed in various types of cells, including pancreatic cancer, melanoma and Hela cells^22^,^23^,^24^,^25^. And recent studies also show that nsPEFs can effectively eliminate murine melanoma and murine basal cell carcinoma *in vivo*^29^,^30^,^31^,^32^. It was reported that MAPK, GCN2 and PERK pathways are involved in the biological effects induced by the nsPEFs^33^,^34^.

Despite of its extensive applications in life sciences, reports regarding the effects and relevant mechanisms of nsPEFs on proteins remain limited and elusive^35^. Using a simulation model, myoglobin was shown to undergo a fast unfolding transition (occurring within 100−200 ps) under a 10^9^ V/m electric (E) field, which in turn caused the denaturation^36^. Several other studies also presented the evidence that E and electromagnetic (EM) fields can render major changes in protein’s secondary structure and disruption of hydrogen bonds associated with protein charged residues^37^,^38^,^39^. Here, we have demonstrated the inactivation efficacy of ricin toxin by nsPEFs, including both *in vitro* with BEAS-2B cells and *in vivo* experiments with mice. The pathological damages in heart, lung, kidney and stomach in mice resulting from exposure of pure and treated ricin by 10 ns, 100 ns and 300 ns nsPEF treatments were observed. Our work further revealed that certain secondary structures of the ricin toxin underwent transition due to the nsPEFs exposure.

## Material and Methods

### Materials used

#### Ricin toxin

In this work, ricin toxin (RT) was prepared and provided by Institute of Microbiology and Epidemiology, Academy of Military Medical Sciences in Beijing. Raw toxins were extracted from the seeds of castor produced in Yunnan and Xinjiang, China. The crude ricin containing the toxins and agglutinins were further purified by the galactose residues on the affinity matrixes, and they were separated using gel filtration column based on the difference in their molecular weight between the toxins and agglutinins. The molecular weight of RT is approximately 64 kDa. By this way, nearly all the unrelated proteins were removed and the purity of toxins can reach up to more than 95% as observed using 12% sodium dodecyl sulfate polyacrylamide gel electrophoresis (SDS-PAGE).

#### Cell lines and cell culture

BEAS-2B cell lines were provided by Institute of Microbiology and Epidemiology, Academy of Military Medical Sciences in Beijing. All cells were cultured in RPMI-1640 (Gibco, Grand Island, NY, USA) supplemented with 10% fetal bovine serum (FBS; HyClone, Logan, UT, USA), 100 U/ml penicillin and 100 U/ml streptomycin. Cells were maintained in a humidified atmosphere of 5% CO_2_ at 37 °C for further experiments with ricin.

#### Nanosecond pulsed electric fields (nsPEFs) and its application

In this study, a self-made nsPEFs generator based on transmission line circuit as described in a previous study^40^ was used for inactivating ricin with an applied electric field of 30 kV/cm and pulse durations from 10 ns to 300 ns. Waveforms were monitored using a digital phosphor oscilloscope (DPO4054, Tektronix. USA) that was equipped with a high voltage probe (P6015A.Tektronix.USA) and the results are shown in Figure S1 (Supporting Information). The ricin toxin powder was first dissolved in the deionized water to form the ricin solution, and then it was treated with the nsPEFs with pulse durations of 10 ns, 100 ns and 300 ns for 200 pulses, respectively. Cells or mice that were exposed to ricin without the nsPEFs treatment were used as positive control group, and those receiving PBS (GIBCO® PBS) exposure were chosen as negative control group.

### Cell experiment and the structure analysis of ricin

#### Exposure experiments with BEAS-2B cells

BEAS-2B cells were seeded in a 96-well plate with a final volume of 100 μL containing 10^4^ cells per well. The plates were incubated at 37 ^o^C for 24 h, and subsequently pre-determined dilutions of ricin were added to triplicate wells (none was added to the negative control, the nsPEF treated ricin was added as the treatment group, and untreated ricin was added to the positive controls). After incubation for 24 h, the plate was washed three times with PBS, and culture medium with a volume of 240 μL was added into each well. Then 10 μL CCK-8 (Cell Counting Kit-8) was added to each well, and the cells were further incubated at 37 °C for 2 h. Dye intensity was then read on a microplate reader (Sigma, St Louis, MO) at 450 nm. The killing efficiency was calculated according to the equation: Killing efficiency (%) = (absorbency of control-absorbency of treated cells)/absorbency of control × 100.

#### Sodium dodecyl sulfate polyacrylamide gel electrophoresis (SDS-PAGE)

The nsPEFs-treated ricin protein samples, one ricin sample without any treatment (positive control) and the deionized water (negative control) were subjected to SDS-PAGE for analyzing ricin primary structure. The concentration of tested ricin solution was 36 μg/mL. 10 μL of the controls, treated ricin protein sample mixed with loading buffer, and also a protein ladder (10–170 kDa) were added separately and subjected to SDS-PAGE analysis using a vertical electrophoresis equipment (Beijing Liuyi instrument factory) operated at a voltage of 80 for 15 min in the first stage and then at a voltage of 120 for 90 min for the second stage. After the experiment, the gel was taken out from the equipment for staining by the silver stain agent (Beijing ComWin Biotech Co.,Ltd.).

#### Circular dichroism (CD) measurements

The secondary structures (three-dimensional form of local segments of biopolymers) of both the nsPEFs treated and untreated ricin were investigated using circular dichroism (CD) spectrum method to study the inactivation mechanisms of nsPEFs on ricin. Circular dichroism spectra recorded at ricin toxin in a quartz cuvette using a cell with a 10-mm light path from 400 to 200 nm, averaging 9 scans per sample with a scanning speed of 100 nm/min using Jasco J-815 apparatus (JASCO Europe S.R.L, Italy). There were totally four different structures (a-helix, beta-sheet, bet-turn, and random coil) detected for the ricin protein, and this test was repeated for 10 times. The fraction of each secondary structure of the ricin was calculated before and after the nsPEFs treatment.

### *In vivo* experiments and pathological analysis

#### Ricin injection to KM mice

We chose totally 100 KM mice with a weight range of 18–22 g, and randomly assigned them into two different groups. For the first group, 50 KM mice were again distributed into five groups, and each group had ten mice. The group 1 was injected with the ricin dissolved in normal saline (9% sodium chloride solution) with a final concentration of 8 μg/mL as a positive control. The KM mice in groups 2–4 were injected with the same ricin but treated with the nsPEFs for 200 pulses with different pulse durations of 10 ns, 100 ns and 300 ns, respectively. The last group was injected only with normal saline as the negative control. Injection volume of ricin solution (8 μg/mL) for each KM mouse was 0.5 mL. After abdominal cavity injection, all the mice were fed to observe the ricin killing rate. Then the test was repeated again for subsequent anatomy.

#### Ricin inhalation by KM mice

**Figure 1:**
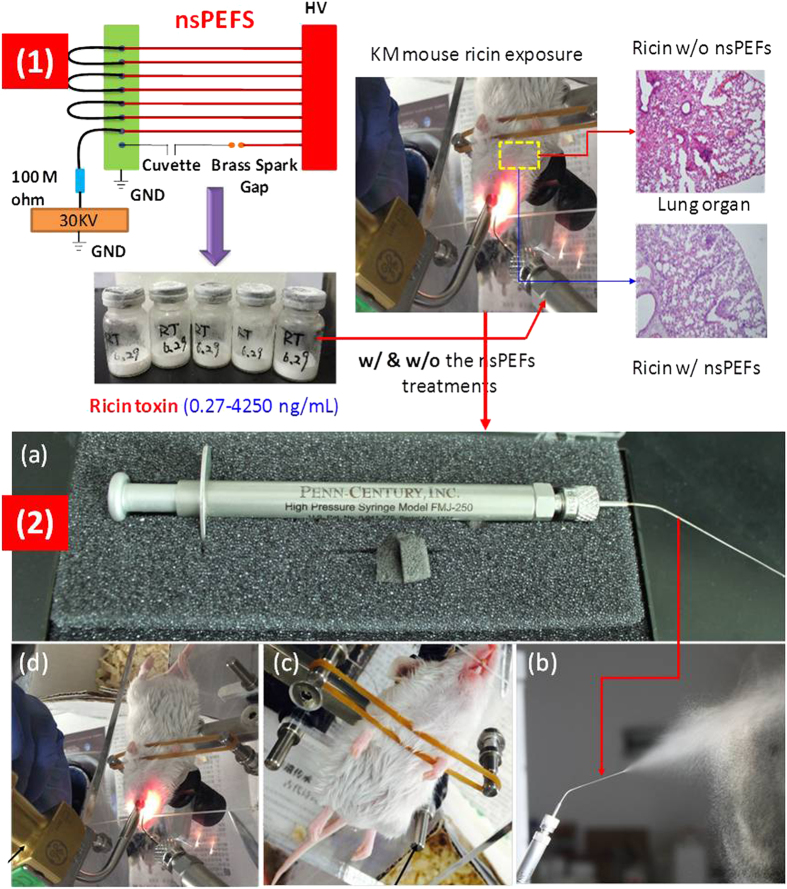
(**1**) Experimental procedures for testing ricin toxicity using mouse model (**2**) The inhalation exposure experiments of KM mouse: (**a**) the ricin aerosolizer utilized in this experiment; (**b**) example of liquid’s aerosolization (the normal saline was used); (**c**) and (**d**) the experimental procedures of anesthetization and inhalation exposure, respectively.

In a practical scenario, ricin stockpile could be hit by a conventional weapon or ricin could be released into the air when loaded with the conventional weapon, thus presenting a serious inhalation risk. Therefore, the study here was designed to investigate its killing efficiency via inhalation when comparing to those inactivated. Figure 1 shows the procedures of ricin toxicity experiments including the aerosolization equipment used and examples of inhalation exposure. For inhalation exposure experiments, the selection criteria and grouping method of KM mouse were the same as the injection group. Before the experiment, the general anaesthesia was carried out on KM mice by 1% pentobarbital sodium salt (shown in Fig. 1 (c)). When the mice totally lost consciousness, a small animal laryngoscope and MicroSprayer® Aerosolizer (Model IA-1C and FMJ-250 High Pressure Syringe, Penn-Century, Inc., USA) (shown in Fig. 1 (a,b)) were utilized to deliver the corresponding ricin solutions with or without direct treatments by the nsPEFs to the pulmonary of every mice (a total of 50) as shown in Fig. 1 (d). The aerosolized ricin droplets size range was estimated to be from 16–22 μm according to the instruction of this device. The delivery volume of ricin solution with a concentration level of 20 μg/mL to every KM mice in each of the treatment group (each group had 10 mice) was 0.5 mL. Following the inhalation exposure, all the KM mice (a total of 50) were raised in the feeding room. Whenever the mice in positive control group (exposure to untreated ricin) were observed to start dying, all the KM mice were euthanized simultaneously in order to conduct anatomy. For studying the eventual ricin killing rate by inhalation, another 35 KM mice were used following the same nsPEFs treatment conditions as described above.

#### Anatomy and histopathological analysis

In this study, we euthanized all the mice involved in the experiments at the time when those mice exposed to the ricin without the nsPEF treatment were dying; and the heart, lung, kidney, liver and stomach of all the groups of mice involved in the experiments were taken, then the organs were subsequently steeped using 4% paraformaldehyde solution for 24 hours. Dehydration and embedding in paraffin conducted subsequently as the preparation of tissue section. In this work, the slice thickness was 5 μm for each organ, and after the cutting they were mounted onto the glass slides and deparaffinized. Finally, all of the resulting slides (a total of 10) were stained with hematoxylin and eosin for analysis according to the standard procedure described^34^. All animal experiments were conducted according to the Guideline for Animal Experiment of the Academy of Military Medical Sciences (Beijing, China), and were approved by the Animal Ethics Committee of the Academy of Military Medical Sciences.

### Statistical analysis

In studying the ricin killing efficiencies, both positive (exposure to untreated ricin) and negative control (no ricin used including saline aerosols and PBS buffer) groups were included. When analyzing differences in the ricin killing efficiencies of BEAS-2B cells and the fractions of ricin’s secondary structures (a-helix, beta-sheet, bet-turn, and random coil) between different treatment groups, independent sample t-test was used via the software SPSS 16.0. A p-value of less than 0.05 indicates a statistically significant difference between different treatment groups.

## Results and Discussion

**Figure 2:**
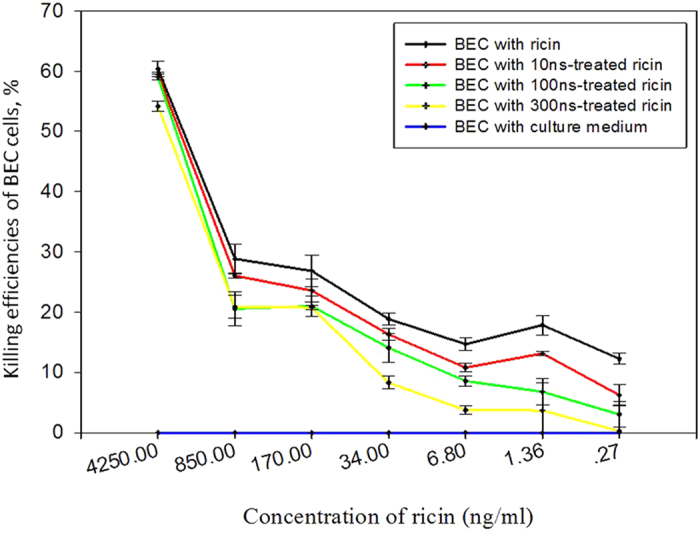
The killing efficiencies of BEAS-2B cells by different levels of ricin (from 0.27 to 4250 ng/mL) with and without the nsPEFs treatment. The respective ricin solutions were treated by nsPEFs with a pulse duration of 10 ns, 100 ns or 300 ns for 100 pluses. The exposure time of BEAS-2B to respective ricin solutions by mixing was 24 hours. Data points represent averages and standard deviations of three different repeats.

Overall, the killing efficiencies of BEAS-2B cells were observed to be up to 60% by ricin at a concentration level of 4250 ng/mL even with the nsPEFs treatment as shown in Fig. 2. As seen from the figure, the killing efficiencies in general decreased with decreasing ricin concentration level. When the test ricin concentration was lowered to 0.27 ng/mL, about 15% of BEAS-2B cells were killed by the untreated ricin. In contrast, no killing was observed if the BEAS-2B cells were mixed with only culture medium as a control. As shown in the figure, after the nsPEFs treatments, the killing efficiencies of the ricin at the same concentration levels in general were lowered, especially pronounced with the 300 ns pulse duration, followed by 100 ns, and 10 ns. For each of ricin concentrations tested, there were statistically significant differences between the treatment effects of nsPEFs with different pulse durations (10 ns, 100 ns, 300 ns) compared to the control without the treatment (p-values <0.05, independent sample t-test).

**Figure 3:**
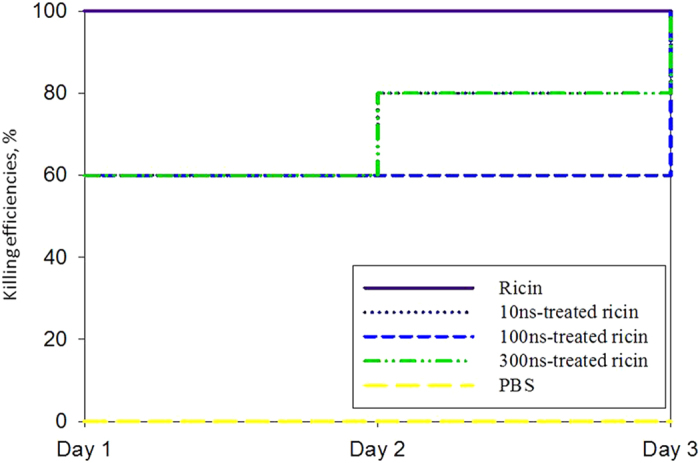
The time-dependent killing efficiencies of KM mice after the intraperitoneal injection of the ricin solutions with and without the nsPEFs treatments. Each group had 10 mice, and a total of 50 mice were involved in this experiment. The injection dose was 4 μg/mouse (concentration of the ricin solution was 8 μg/ml, and totally 0.5 ml was injected for each mouse).

Figure 3 shows the time-dependent killing efficiencies of KM mice after the intraperitoneal injection of 4 μg ricin per mice. Without the nsPEFs treatment, 100% of KM mice were killed upon the 4 μg ricin injection on Day 1 as seen in Fig. 3. In general, 40% of the mice in each group survived the first day when exposed to the ricin treated by the nsPEFs as observed in Fig. 3. No killing was observed when the KM mice were injected with PBS buffer as a control. Regardless of nsPEFs treatment pulse durations, no KM mice survived on Day 3 when injected with the 4 μg ricin treated or not treated by the nsPEFs. For the 10 ns-nsPEFs and 300 ns-nsPEFs treatment groups, the killing efficiencies both increased from 60% on Day 1 to 100% on Day 3. However, for the mouse group exposed to the ricin treated by 10 ns-nsPEFs treatment, the killing efficiencies further increased to 80% on Day 2, and up to 100% on Day 3. The results with mouse experiments were different from those conducted using the BEAS-2B cells with respect to the inactivation effects of100 ns and 300 ns pulse durations. The difference was likely due to different killing mechanisms between the BEAS-2B cells and mice.

**Figure 4:**
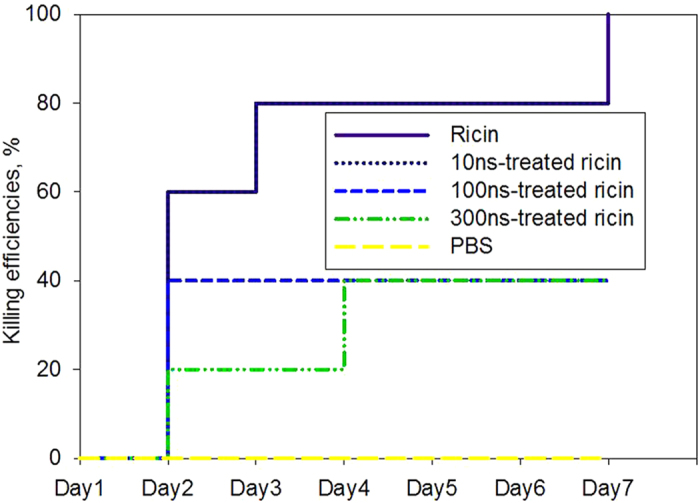
The time-dependent killing efficiencies of KM mice when exposed to airborne ricin of 10 μg per mouse under three different nsPEFs treatment conditions. During the inhalation exposure test, 0.5 mL ricin solution of 20 μg/mL was aerosolized using the MicroSprayer® Aerosolizer into the lung of every mouse. Each group had 10 mice, and a total of 50 mice were involved in the experiment.

The time-dependent killing efficiencies of KM mouse by airborne ricin under different treatment conditions were shown in Fig. 4. For the negative control-PBS buffer, no killing was observed. For the ricin without the nsPEFs treatment, no killing was observed on Day 1, however 60% of the mice died and further increased to 80% on Day 3 and up to 100% killing on Day 7 as shown in Fig. 4. For airborne exposure, it seems 10 ns-nsPEFs treatment did not affect the killing efficiencies of inhaled ricin compared to no nsPEFs treatment. Nonetheless, on Day 7 the killing efficiency was observed up to 100% for ricin without the nsPEFs treatment, but about 80% was observed for ricin treated with the 10 nsPEFs. For the 300 ns nsPEFs treatment, 80% of the mice survived the ricin exposure on Day 2, and further decreased to 60% on Day 4 and then after. In contrast, for the 100 ns nsPEFs treatment 60% of the mice survived the treatment throughout the 7-day time period as seen in Fig. 4. Similar to the BEAS-2B cell experiments, the 300 ns-nsPEFs treatment showed the best inactivation efficiency to the ricin of 20 μg/mL, while the 10 ns-nsPEFs treatment did not result in a visible effect on the ricin toxicity.

Results from Figs 3 and 4 suggest that the intraperitoneal injection of ricin with similar dose levels would induce higher mortality than the inhalation exposure. However, the mortality rate of inhaling ricin was shown to be higher than ingestion^7^. Therefore, the intraperitoneal injection of ricin might represent the highest mortality risk, followed by inhalation and ingestion routes. Through the *in vitro* and *in vivo* experiments, we can see that after the treatments by the ns-PEFs, the death time of BEAS-2B cells and KM mice could be delayed efficiently (at least one day compared to the experiments with ricin without the nsPEFs treatment) for both injection and inhalation tests. Generally, the time-dependent killing efficiencies also decreased in response to the nsPEFs treatment. These results indicated that the nsPEFs can efficiently inactivate ricin under certain conditions, for example, the 300n-nsPEFs treatment for inhalation experiments. In previous studies, it was shown that increasing pulse duration increased the inactivation of *E. coli*^41^. It was also found that after exposure to 20 pulses of 0.7 ms at 6.2 kV/cm, the activity of the protease from *Pseudomonas fluorescens* was reduced by 30%^42^. The nsPEFs-induced inactivation of lipase up to 60% was established only at an energy input of exceeding 200 J/mL^43^. The energy input per pulse (W [J/ml]) was determined using the following equation (1) as proposed by Wouters, Alvarez, and Raso (2001)^44^:


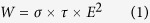


where 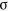 is the conductivity (S/m), 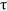 is the pulse duration (s) and *E* the electric field strength (V/cm). Therefore, with the same field intensity and number of pulses, the energy input was determined by the pulse duration. As indicated, the energy input also depends on the conductivity of the medium receiving the nsPEFs. By calculation, the energy input per pulse of the 300 ns-nsPEFs treatment here was 30 times higher than 10 ns-nsPEFs treatment. This helps explain why the 300 ns-nsPEFs treatment showed better inactivation efficiency to ricin than 10 ns-nsPEFs and 100 ns-nsPEFs treatment as demonstrated in airborne exposure experiments with mouse and also in the BEAS-2B cell experiments. Nonetheless, different phenomenon was observed for the ricin injection experiments with mouse. This difference could be to different exposure route and also possibly difference in mechanisms inducing toxicity to cells and mice by the ricin toxin.

**Figure 5:**
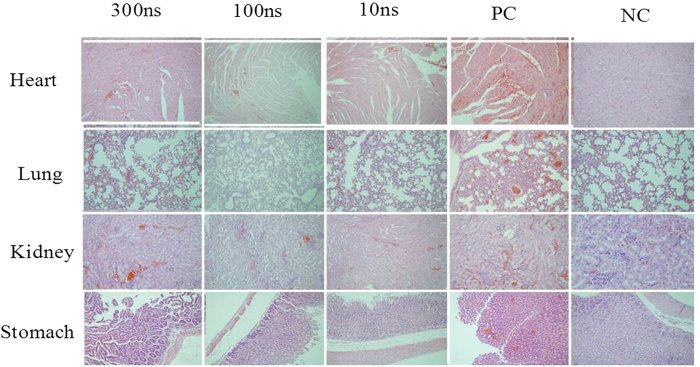
Pathological observations of damages (e.g., the red spots) to four different organs (heart, lung, kidney, stomach) from KM mice which were directly injected with ricin (4 μg/mouse) that was treated with the nsPEFs with different pulse durations (10 ns, 100 ns and 300 ns) for 200 pulses. PC refers to the KM mouse group injected with 4 μg ricin per mice directly without the nsPEFs treatment, and NC refers to the KM mouse group injected with only normal saline.

The time-dependent killing mechanisms of KM mice by the ricin injection were further studied by pathological experiments after different exposure and treatment conditions as shown in Fig. 5. Overall, there were damages (e.g., the red spots shown in the figure) were observed in different organs of KM mice when injected with the ricin with and without the nsPEFs treatments as shown in Fig. 5. For the PC group (ricin without the treatment), pronounced damages were found to heart, lung, kidney and stomach, as shown in Fig. 5 with the appearance of eosinophilic proteinaceous fluid (edema) and plentiful fibrin strands mixed with large amount of degenerated and viable neutrophils. Additionally, for the positive control group fewer numbers of lymphocytes, plasma cells, and macrophages were observed. It was demonstarted that 100 ns and 300 ns nsPEFs had better detoxifying efficiencies on ricin toxin than 10 ns one as manifested by pathological observations in Fig. 5. Compared to the PC group, the free erythrocytes (hemorrhage) reduced drastically in heart, lung, kidney and stomach in the 100 ns-nsPEFs and 300 ns-nsPEFs treated groups. In addition, the ricin toxin was observed to cause serious damages to lung and kidney via the intraperitoneal injection as observed in Fig. 5. Here, in addition to congestion, hemorrhage and inflammatory cell infiltration observed for all organs in the mice challenged with the ricin, the intervals of pulmonary were additionally observed to broaden and the structure of kidney tubules became fuzzy. For the negative control group, we did not observe any damages to the mice’s organs.

**Figure 6:**
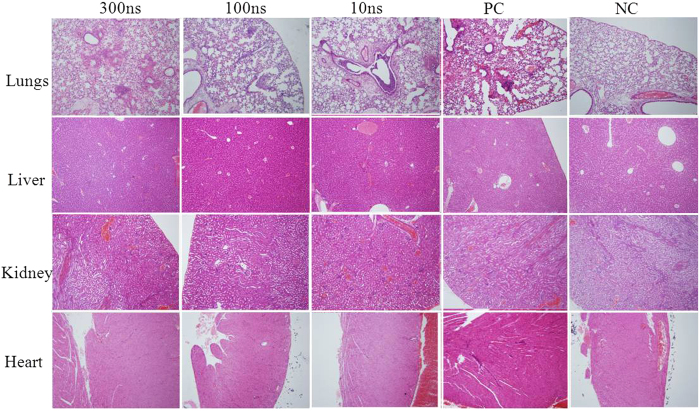
Pathological observations of damages (e.g., the red spots especially for the lung) to four organs (lung, liver, kidney and heart) from KM mice after ricin aerosol exposure (10 μg/mouse) with and without the nsPEFs treatment with different pulse durations (10 ns, 100 ns and 300 ns) for 200 pulses. PC refers to the KM mouse group exposed to 10 μg/mouse ricin toxin via inhalation without treatment, and NC refers to the KM mouse group exposed to only saline aerosols.

However, the pathology results of inhalation exposure were slightly different from those observed from the injection ones. As seen from Fig. 6, the inhalation exposure of ricin toxin (10 μg/mouse) with and without the nsPEFs treatment caused pronounced injuries to the lungs of KM mice; however, in contrast to the injection exposure the damages to other organs (liver, heart, and kidney) were minor as observed in Fig. 6. As it is shown in PC group in Fig. 6, the fibrin, edema fluid, and inflammatory infiltrate expanded the alveolar septa. Besides, in the alveolar spaces more cellular debris and degenerated neutrophils appeared, accompanying with the edema fluid. In the multifocal necrosis areas of pulmonary, the alveolar epithelial cells and architectures were observed to be partly or completely damaged. Also, it was observed that neutrophils, lymphocytes, and macrophages accompanying edema fluid appeared in or around the perivascular, peribronchial, and peribronchiolar areas. It can be clearly seen that the treatment by the 10 ns-nsPEFs did not result in the inactivation of ricin because severe damages to the lung occurred to the group. It has been reported by many studies that the severe lesions in the lower respiratory tract would occur in the *vivo* experiments of ricin toxin’s inhalation exposure on rhesus macaques, Sprague-Dawley rats, and BALB/c mice^45^,^46^,^47^,^48^,^49^,^50^. According to a previous study, the severe pulmonary lesion leading to the impediment of oxygen exchange was the immediate cause of death of living bodies^49^. As it is demonstrated in Figs 5 and 6, the ricin treated by the nsPEFs with 100 ns, 300 ns and 10 ns pulse duration, respectively, can effectively reduce the damage to lungs of KM mice. However, the 10s-nsPEFs treatment had the least ricin inactivation effect. This was due to the lower energy input per pulse as indicated by the equation described above.

**Figure 7:**
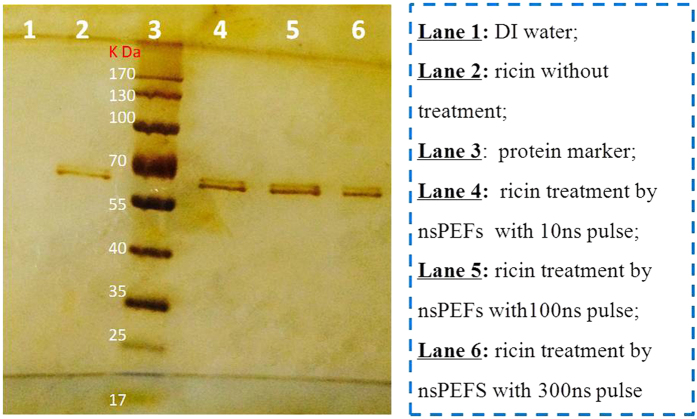
The primary structure of ricin (36 μg/ml) protein detected by SDS-PAGE with and without the nsPEFs treatments (10 ns, 100 ns, and 300 ns). Each group was treated for 200 pulses.

**Figure 8:**
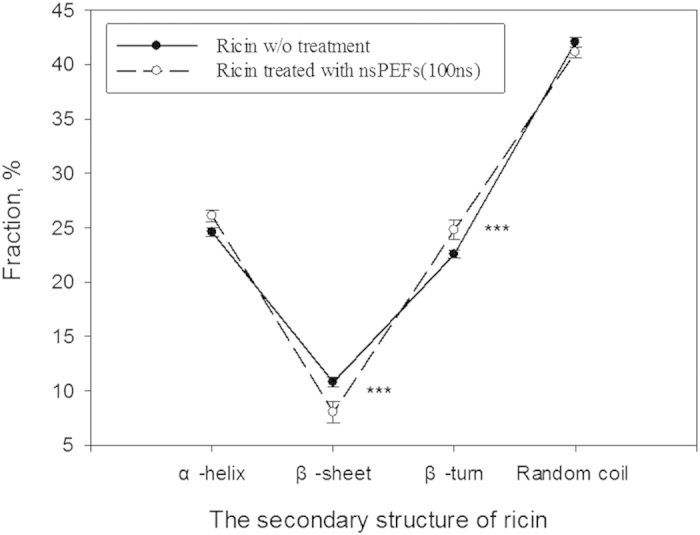
The fractions of ricin protein’s secondary structures (a-helix, beta-sheet, bet-turn, and random coil) detected by the circular dichroism spectrum before and after 100 ns nsPEFs treatment for 200 pulses. The concentration of ricin used was 0.5 mg/mL, and data points represent averages and standard deviations from 10 independent repeats; *** indicates a statistically significant difference (independent sample t-test): p-value = 0.034 for β-sheet and p-value = 0.021 for β-sheet turn secondary structure.

To further investigate the detoxifying mechanisms of the nsPEFs treatment on ricin, SDS-PAGE experiments were carried out and the results are shown in Fig. 7. As observed from the figure, even after the nsPEFs treatments (10 ns, 100 ns, and 300 ns) the target bands for ricin were still present for the three different treatments. The SDS-PAGE results in Fig. 7 indicate that the length of ricin protein’s peptide chain did not break under high exogenous electric pulses tested in this work. Accordingly, the circular dichroism was utilized to study if there is a change in the secondary structure of ricin protein. As shown in Fig. 8, the fraction of β-sheet decreased while that of β-turn increased in ricin’s peptide chains upon the nsPEFs treatment. There were statistically significant differences in the fractions of the ricin’s secondary structures (beta-sheet and beta-turn) (p-value = 0.034 and 0.021, respectively) before and after the nsPEFs treatment. However, no statistically significant differences were observed for the fractions of other two structures: a-helix and random coil. These data suggest that beta-sheet structure might be responsible for the toxicity of the ricin. With the nsPEFs exposure, beta-sheet structure might have transitioned to beta-turn form.

As a type 2 ribosome-inactivating protein, ricin toxin consists two polypeptide chains: RTA (32 kDa) and RTB(34 kDa) which are combined via disulfide bridge^51^,^52^,^53^. Overall, there are three structural domains in ricin chain A (the toxic component) which approximately arranges 50% of the polypeptide into alpha-helices and β—sheets^54^. In contrast, the structure of ricin chain B lacks alpha-helices or beta-sheets^55^. Here, we have observed a decline in the fraction of β-sheets and an increase in β-turn of the ricin toxin in response to the nsPEFs treatment. Such a change upon the nsPEFs treatment might have directly resulted in ricin’s lower toxicity observed using BEAS-2B cells and mice. This structure transition resulted from the interaction between the ricin’s secondary structures and the nsPEFs. It was previously reported that polypeptide chains of protein could be affected by an external electric field^56^,^57^. Zhao *et al.* revealed that the nsPEFs could trigger an augment in the dielectric constant of protein, thus inducing the unfolding of polypeptide, and then the secondary and tertiary structures of protein would vary^58^. In another study, Marracino *et al.* simulated the exposure of myoglobin in pulsed and static electric fields, and they observed a fast transition between two different secondary structures: myoglobin-folded and unfolded states^36^. To our best knowledge, our study is the first one to report the effects of the nsPEFs on ricin toxin’s toxicity, primary and secondary structures. The ricin treatment efficacies by the nsPEFs have been demonstrated using *in vitro* and *in vivo* experiments. The results here not only provide a practical ricin toxin threat combat solution, but also identify the new mechanisms of detoxifying the ricin toxin. In addition to combating the bio-terrorism activities, the developed technology might be immediately useful to treating wastewater when manufacturing castor oil. Future tests with stronger electric field could be explored to further increase the ricin inactivation efficiencies.

## Additional Information

**How to cite this article**: Wei, K. *et al.* Inactivation of Ricin Toxin by Nanosecond Pulsed Electric Fields Including Evidences from Cell and Animal Toxicity. *Sci. Rep.* **6**, 18781; doi: 10.1038/srep18781 (2016).

## Supplementary Material

Supplementary Information
