# Supplementary material for: Inactivation of Ricin Toxin by Nanosecond Pulsed Electric Fields Including Evidences from Cell and Animal Toxicity
Source: Sci Rep. 2016 Jan 5;6:18781. doi: 10.1038/srep18781 (PMC4700442; doi:10.1038/srep18781)
Supplement: Supplementary Information [file srep18781-s1.doc]

Inactivation of Ricin Toxin by Nanosecond Pulsed Electric Fields Including Evidences from Cell and Animal Toxicity

Kai Wei^1,#^,, Wei Li^2,#^, Shan Gao^3^, Bin Ji^3^, Yating Zang^3^, Bo Su^2^,

Kaile Wang^2^, Maosheng Yao^1^,*, Jue Zhang^2,4^,*, and Jinglin Wang^3^,*

^1^State Key Joint Laboratory of Environmental Simulation and Pollution Control, College of Environmental Sciences and Engineering, Peking University, Beijing 100871, China

^2^ Academy for Advanced Interdisciplinary Studies, Peking University, Beijing 100871, China

^3^ Institute of Microbiology and Epidemiology, Academy of Military Medical Sciences, Beijing 100071, China

^4^ College of Engineering, Peking University, Beijing 100871, China

Revision submitted to

Scientific Reports

____________________________________________________

*Corresponding authors:

Maosheng Yao, yao@pku.edu.cn, +86 010 6276 7282

State Key Joint Laboratory of Environmental Simulation and Pollution Control, College of Environmental Sciences and Engineering, Peking University, Beijing 100871, China

Jue Zhang, zhangjue@pku.edu.cn, +86 010 62755036

Department of Biomedical Engineering, College of Engineering, Peking University, Beijing 100871, China

Jinglin Wang, [wjl0801@vip.163.com](mailto:wjl0801@vip.163.com), +86 010 66948643

Institute of Microbiology and Epidemiology, Academy of Military Medical Sciences, Beijing 100071, China

#: K. Wei and W. Li contributed equally to the work

Beijing

2015-11-10

Supporting Information


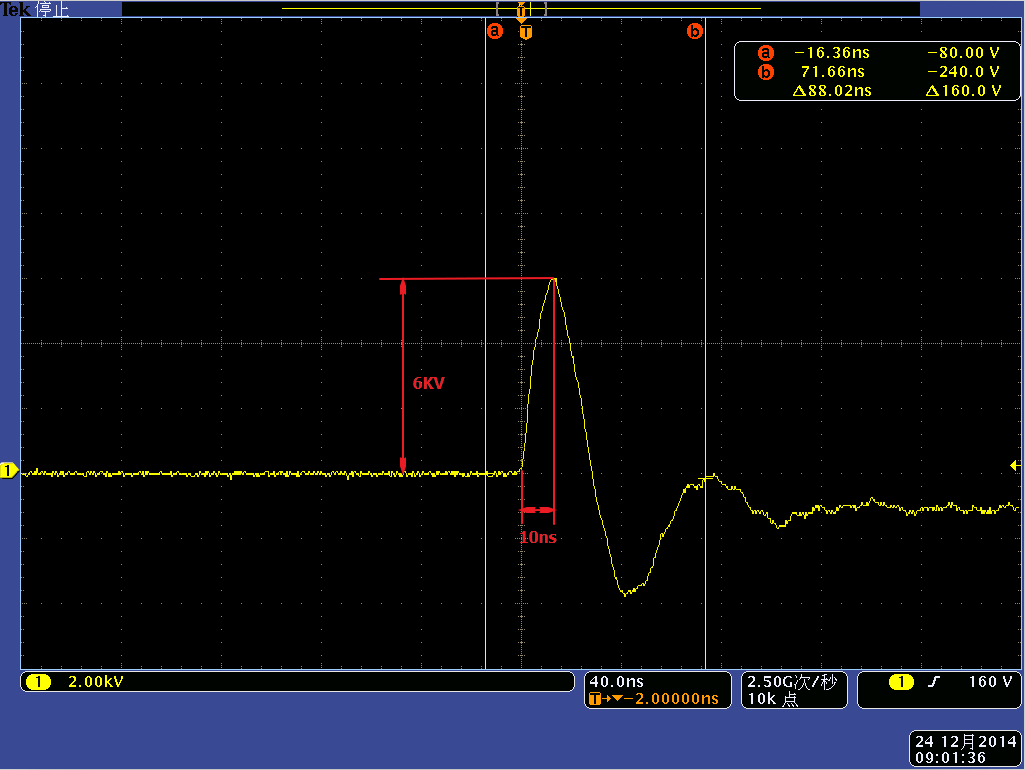


(a)


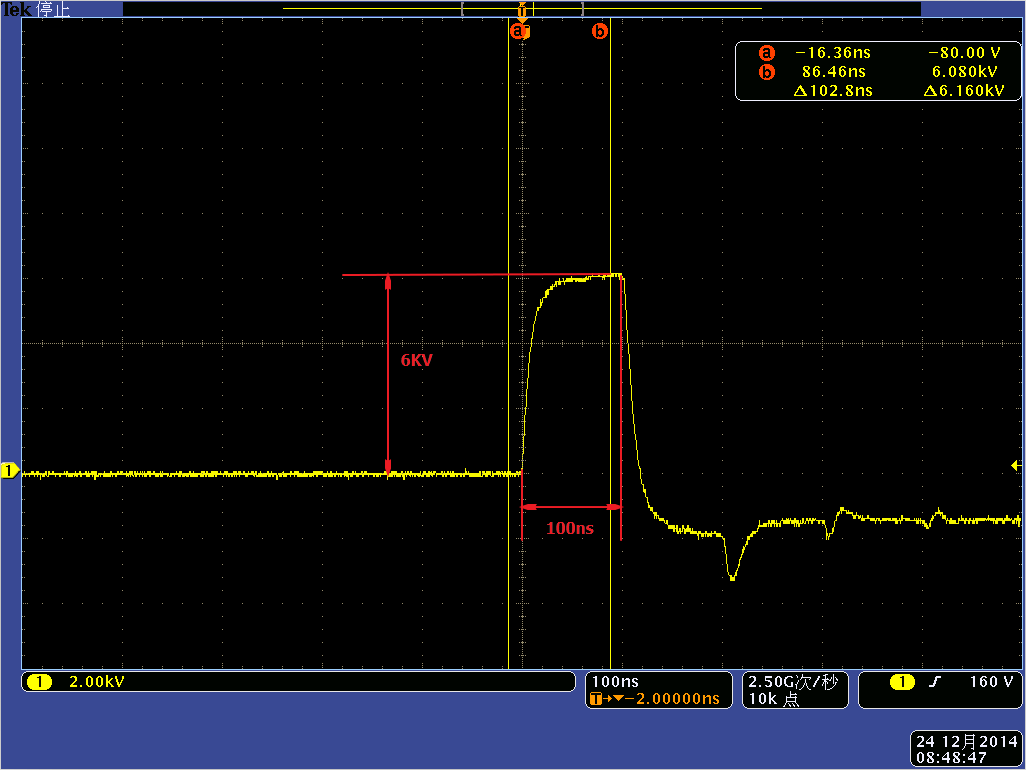


(b)


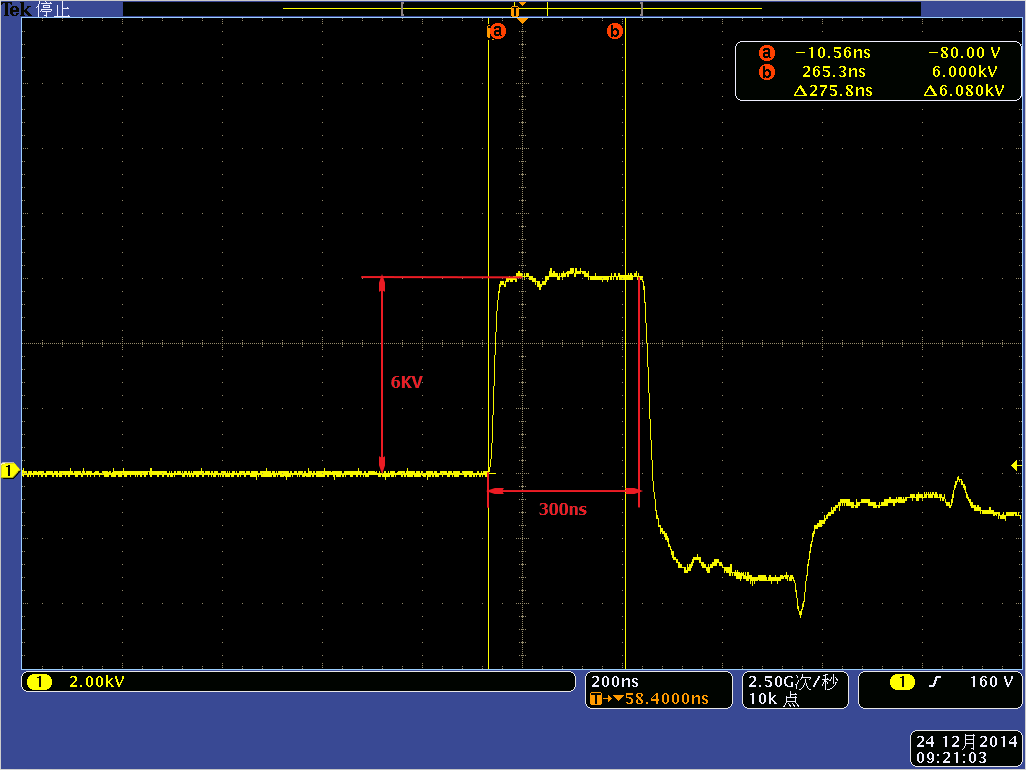


(c)

**Figure S1**. The typical waveforms of nsPEFs treatment of ricin, the horizontal axis shows pulse duration in scales of nanoseconds, the vertical axis represents voltage, each bar equals 2KV. A total voltage of 6KV is applied on the electrodes with a distance of 2mm, so the electric field between the electrodes is 30 kV/cm. (a) nsPEFs treatment with a pulse duration of 10ns; (b) nsPEFs treatment with a pulse duration of 100ns ; (c) nsPEFs treatment with a pulse duration of 300ns. The pulse duration was represented by wave crest width.
